# Galectin-3 in Cardiovascular Health: A Narrative Review Based on Life’s Essential 8 and Life’s Simple 7 Frameworks

**DOI:** 10.3390/cimb47050332

**Published:** 2025-05-06

**Authors:** Adrian Martuszewski, Patrycja Paluszkiewicz, Rafał Poręba, Paweł Gać

**Affiliations:** 1 Department of Environmental Health, Occupational Medicine and Epidemiology, Wroclaw Medical University, Mikulicza-Radeckiego 7, 50-345 Wrocław, Poland; adrian.martuszewski@student.umw.edu.pl; 2 Department of Neurology, Specialist Hospital in Walbrzych, 58-309 Wałbrzych, Poland; 3 Department of Emergency Medical Service, Wroclaw Medical University, Bartla 5, 50-367 Wrocław, Poland; 4 Department of Biological Principles of Physical Activity, Wroclaw University of Health and Sport Sciences, 51-612 Wrocław, Poland; 5 Centre of Diagnostic Imaging, 4th Military Hospital, Weigla 5, 50-981 Wrocław, Poland

**Keywords:** environmental factors, cardiovascular diseases, public health, healthy lifestyle, inflammation, galectin-3, fibrosis, molecular mechanisms, health status, biomarkers

## Abstract

Gal-3, also known as galectin-3, a lectin that binds β-galactosides, has gained attention as a novel biomarker and pathophysiological mediator in cardiovascular disease, where it contributes to inflammation, fibrosis, metabolic dysregulation and cardiac remodeling. This narrative review, developed following SANRA (Scale for the Assessment of Narrative Review Articles) guidelines, aims to integrate current clinical and experimental findings on gal-3 into the American Heart Association Life’s Simple 7 (LS7) and Life’s Essential 8 (LE8). By thematically organizing our review across modifiable domains of cardiovascular health, including glucose regulation, lipid metabolism, physical activity, blood pressure, diet, sleep, tobacco use, and body weight (BMI, body mass index), we highlight the role of gal-3 in linking environmental, behavioral and molecular risk factors to cardiometabolic outcomes. Particular attention is given to the oxidative stress, inflammatory–fibrotic axis, and immune activation as mechanistic pathways underlying gal-3-associated cardiovascular damage. We also discuss its relevance to public health and prevention, considering gal-3’s potential for early risk stratification, monitoring lifestyle interventions and personalized prevention strategies. This review bridges molecular mechanisms with clinical and public health relevance, particularly in the context of environmental and lifestyle-related cardiovascular risk.

## 1. Introduction

Recent perspectives highlight the growing importance of early biomarker identification and long-term lifestyle monitoring in cardiovascular disease (CVD) prevention, particularly arterial hypertension (AH), in relation to environmental exposures and occupational factors [1].

Large-scale population studies, such as the Framingham Heart Study and PREVEND, have established galectin-3 (gal-3) as a biomarker associated with an elevated risk of heart failure (HF), cardiovascular mortality, and all-cause mortality [2,3]. Although the prognostic role of gal-3 is known, there is still a lack of clear integration of this knowledge with current cardiovascular health (CVH) assessment scales—Life’s Simple 7 (LS7) and Life’s Essential 8 (LE8).

Gal-3 performs a range of functions both within the cell and outside of it. It is involved, among other things, in the regulation of cell adhesion, intercellular signaling, immune response, inflammatory processes, fibrosis, and apoptosis [4]. Elevated gal-3 concentrations have been linked to adverse cardiac outcomes and poorer prognosis, supporting its proposed role in cardiovascular risk stratification, assessment of myocardial fibrosis, and monitoring therapeutic response [5].

In this narrative review, the possible role of gal-3 as a marker of CVH, whose definition is included in the LS7 and LE8 scales, is discussed. Furthermore, the structure of gal-3, its key biological functions, and the associated molecular mechanisms are addressed.

### 1.1. The Place of Galectin-3 in the Guidelines

The role of gal-3 has been included in cardiology guidelines as a complementary biomarker. Since 2010, the U.S. FDA has approved the use of galectin-3 as a complementary prognostic marker in chronic HF, to be interpreted alongside clinical evaluation. The guidelines of the ESC indicate the need for further studies on the clinical utility of gal-3 [6]. Both the European (ESC) and American (ACC/AHA/HFSA) HF guidelines mention gal-3 (alongside sST2) as a so-called ‘new’ prognostic biomarker, albeit with a limited recommendation (class IIb) [7]. This means that its measurement may be considered in certain situations for risk assessment (e.g., risk of hospitalization or death in HF), but routine use is not yet the standard of care. Due to its pleiotropic nature, gal-3 is a promising target for biomedical diagnostic and therapeutic research [8,9,10].

### 1.2. Ideal Cardiovascular Health

In 2010, the AHA defined ideal CVH through seven parameters encompassing ideal behaviors and health factors. Later, in an educational campaign, the AHA referred to these recommendations as LS7, which included: tobacco smoking, diet, physical activity, body mass index (BMI), total cholesterol, blood pressure (BP), and glycemia. Each baseline CVH measure was classified as ideal, intermediate, or poor. The mentioned healthy diet components are part of the Dietary Approaches to Stop Hypertension [11]. Table 1 presents the AHA LS7 as the ideal CVH definition.

In 2022, LS7 was expanded to LE8 by adding sleep health as the eighth element. Sleep was assessed based on the reported average number of hours of sleep per day. The following question was added: “On average, how many hours of sleep do you get per night?” and, when available, data from sleep-monitoring devices were also taken into account. Sleep health was evaluated according to the LE8 scale, where the optimal sleep range for adults (7–< 9 h) was assigned the highest score (100 points). Deviations from this range resulted in a lower score: 90 points for 9–< 10 h, 70 points for 6–< 7 h, 40 points for 5–< 6 h or ≥ 10 h, 20 points for 4–< 5 h, and 0 points for < 4 h of sleep per day [16,17]. LE8 is considered a better predictor of stroke occurrence [18]. LE8 and LS7 parameters are used to assess so-called ideal CVH, and meeting them is associated with a lower risk of cardiovascular events. LS7 and LE8 focus on modifiable risk factors. Meanwhile, gal-3 is not directly included in these scales. It is a biological indicator potentially associated with the consequences of neglecting the aforementioned factors.

## 2. Materials and Methods

Our narrative review was prepared following the SANRA (Scale for the Assessment of Narrative Review Articles) criteria, ensuring originality and scientific rigor [19]. A literature review was conducted in accordance with the guidelines for preparing narrative reviews (taking into account the SANRA quality criteria). The focus was primarily on the most recent available publications from the last 10–15 years (mainly 2010–2024). The PubMed/MEDLINE, Embase, Google Scholar, and Scopus databases, as well as the Web of Science search engine, were searched.

Primarily original works (clinical studies—both randomized and observational cohort, cross-sectional), meta-analyses, and systematic reviews, as well as society guidelines and recognized narrative reviews concerning gal-3 were included in the review. Studies on human populations were preferred. Focus was directed toward research assessing gal-3 concentrations in relation to cardiovascular risk, the biological importance of gal-3, and its prospective diagnostic and therapeutic value.

Anecdotal reports, single case descriptions (unless they illustrated a unique mechanism), conference abstracts not supported by a full text, and publications lacking scientific merit (e.g., letters to the editor without data) were excluded. Works written in languages other than English or Polish were also excluded. Studies conducted solely on animal or cell models were not considered if they did not significantly contribute to understanding gal-3 in humans.

The search was conducted separately for each subtopic of the review, combining keywords related to gal-3 and the given issue. Language filters (primarily English-language works) and time filters (limited to the last several years) were applied in the databases. A combination of MeSH terms and free-text synonyms for ‘galectin-3’ and each LS7/LE8 domain was used in the databases.

A search was conducted, initially identifying over 3300 records. After screening titles, abstracts, and full texts, 68 articles were selected for narrative synthesis, structured according to the LS7 and LE8 domains. Studies were included if they met the following criteria: (1) original peer-reviewed research; (2) conducted in human populations; (3) reporting circulating or tissue levels of galectin-3; (4) addressing cardiovascular, metabolic, or neurological outcomes related to LS7/LE8 domains; and (5) published in English or Polish. Preclinical studies (in vitro or animal models) were cited selectively only when they provided critical mechanistic insights that were not available from human studies, and their nature was explicitly indicated in the text. This review was designed as a narrative synthesis; therefore, no formal risk of bias assessment was performed.

The reference lists of the identified articles were also reviewed for additional sources (so-called snowballing). As a result of this process, several dozen items were collected, from which the most relevant and highest-quality evidence was selected for discussion in this review.

In reporting the results of the cited primary studies, a simplified approach to statistical significance was applied. *p*-values are presented as either *p* < 0.05 or *p* < 0.001, depending on the level of significance reported in the original sources (except tables). This distinction aims to improve clarity and allows for an approximate assessment of the statistical strength of the findings, without introducing excessive numerical precision. *p*-values were not interpreted as indicators of effect size or clinical relevance [20,21,22]. Where available, effect estimates (e.g., *OR*, *HR*, or mean differences) are also provided along with confidence intervals, which more accurately convey the precision and potential clinical importance of the findings

In accordance with principles of transparency and methodological clarity, this narrative review was prospectively registered on the Open Science Framework under an open-ended registration [23].

## 3. Properties of Galectin

### 3.1. Structure and Biological Functions of Galectin-3

Gal-3 belongs to the family of lectins that bind β-galactosides, characterized by a unique structure and diverse biological functions, including the regulation of inflammatory processes, fibrosis, and cell adhesion. Gal-3 is present both inside cells and in the extracellular space, performing functions related to adhesion, cell migration, and the regulation of the immune response [8,10].

Gal-3 possesses a carbohydrate recognition domain of approximately 130 amino acids responsible for binding β-galactosides, along with an unstructured N-terminal domain that facilitates oligomerization [4,5,24]. In solution, galectin-3 typically exists as a monomer but can form pentamers upon interaction with polysaccharides [9]. It is localized broadly within cells, being present in the cytoplasm, nucleus, and extracellular space [5].

In the extracellular environment, gal-3 also participates in angiogenesis and regulates leukocyte activity, influencing the course of the immune response. It can both activate leukocytes and exert anti-inflammatory effects by inducing the apoptosis of T lymphocytes [4,5].

Given these features, gal-3 has been increasingly studied for its diagnostic relevance in cardiovascular diseases and as a target for antifibrotic therapies. Table 2 presents the characteristics and functions of gal-3.

### 3.2. The Role in Inflammation and Fibrosis

Galectin-3, secreted by activated macrophages, mediates inflammatory processes and tissue remodeling, particularly by promoting myocardial fibrosis and increasing cardiac stiffness and dysfunction [5]. In other organs, such as the kidneys, liver, and lungs, it also contributes to the progression of fibrosis and inflammation, and its elevated concentrations are associated with the worsening of these processes, for example, in diabetic nephropathy or idiopathic pulmonary fibrosis [7,47]. Gal-3 also plays a significant role in cancer progression by promoting metastasis [48].

In an inflammatory environment, gal-3 acts not only as a macrophage-regulating factor but also as a DAMP/alarmin, signaling the presence of tissue damage or infection. However, its immunomodulatory effects are tissue context-dependent—in some settings, it may support the immune response, while in others, it may exert immunosuppressive effects.

Because of its essential function in tissue remodeling, gal-3 is considered a potential signal of inflammatory and fibrotic activity, notably in heart failure, where elevated blood concentrations may point to active remodeling [5]. Table 3 presents the role of gal-3 in inflammation and fibrosis.

### 3.3. Molecular Mechanisms

Galectin-3 exerts its functions through various molecular mechanisms, stemming from its ability to bind sugars as well as interact with proteins. These mechanisms underlie the previously described processes of inflammation and fibrosis, integrating the activation of the mentioned pathways [49,50]. A specific binding pocket in the carbohydrate recognition domain (CRD) recognizes β-galactosides present in glycoproteins and glycolipids. This binding relies on weak interactions (hydrophobic, hydrogen bonds), making it reversible; sugar ligands, such as lactose, can effectively block it. Many functions of gal-3 result from its cooperation or competition with other galectins, such as galectin-1, which influences the regulation of biological responses under various conditions [4,55]. Gal-3 interacts not only with sugars but also with various cytoplasmic and nuclear proteins. For example, through the NWGR motif (Asparagine-Tryptophan-Glycine-Arginine motif) within the CRD, it binds to Bax and Bcl-2, protecting the cell from apoptosis [8,28]. In the cell nucleus, it cooperates with SR proteins (serine/arginine-rich proteins), forming part of spliceosome complexes and regulating pre-mRNA processing. Additionally, gal-3 binds to PI3K, Ras, and FAK kinases, influencing cellular decisions regarding proliferation and migration. Despite lacking a classical signal sequence, galectin-3 is likely secreted via exosomes and microvesicles. In the extracellular environment, it acts in an autocrine and paracrine manner, activating glycan-dependent receptors [8].

**Figure 1:**
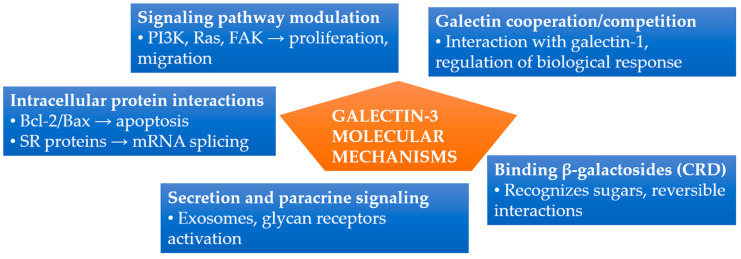
Summary of molecular mechanisms of galectin-3 activity. Gal-3 functions through carbohydrate binding, intracellular protein interactions, signaling pathway modulation, extracellular secretion, and interactions with other galectins. Abbreviations: CRD, carbohydrate recognition domain; FAK, focal adhesion kinase; PI3K, phosphoinositide 3-kinase; SR proteins, serine/arginine-rich proteins.

The main molecular mechanisms described above are briefly summarized and illustrated in Figure 1. Galectin-3’s CRD binds β-galactosides reversibly [4]. Intracellularly, gal-3 modulates apoptosis via Bax/Bcl-2 interaction and regulates mRNA splicing through SR proteins [8,28]. It also interacts with PI3K, Ras, and FAK kinases, affecting proliferation and migration. Extracellular secretion occurs via exosomes, mediating paracrine signaling through glycan receptor activation [8]. Additionally, gal-3 cooperates or competes with galectin-1, modulating biological responses [4,55].

Galectin-3 combines in its structure unique features (a single lectin domain with an attached disordered oligomerizing tail), which translate into a wide range of biological functions. Thanks to its ability to recognize sugars and form interaction networks, gal-3 influences cell adhesion, receptor organization on the membrane, and signal transduction. At the same time, through direct binding to intracellular proteins, it regulates key cellular processes such as apoptosis and gene expression. The multifunctionality of gal-3 makes it a crucial player in maintaining homeostasis, and disruptions in its expression or activity contribute to the development of numerous diseases. Therefore, gal-3 is the subject of extensive research regarding its role in the diagnosis and therapy of inflammatory, fibrotic, and neoplastic conditions.

### 3.4. Galectin-3 as a Biomarker in Cardiology

Galectin-3 contributes to key inflammatory and fibrotic pathways implicated in CVD progression. The most extensive data concern its use in HF, where elevated gal-3 concentrations correlate with increased fibrosis, worse prognosis, and higher mortality, with a hazard ratio of 1.38 for all-cause mortality [7]. Similarly, in acute coronary syndromes, gal-3 acts as a marker of inflammation and cardiac remodeling, significantly elevated in STEMI patients compared to controls (10.6 ng/mL vs. 5.5 ng/mL, respectively) [56]. In atrial fibrillation, gal-3 is recognized as an indicator of atrial fibrosis, associated with increased atrial fibrillation risk (*OR* = 1.45) [57]. Moreover, gal-3 concentrations correlate with left ventricular hypertrophy in AH, indicating its role in the progression of structural cardiac changes [58]. Finally, in patients with aortic stenosis, elevated gal-3 concentrations correlate with left ventricular dysfunction; however, current data on its prognostic utility in this condition remain inconclusive [59].

Galectin-3 remains under investigation as a prognostic and therapeutic marker, although its routine clinical use requires further validation.

### 3.5. Galectin-3 Inhibitors

Given the role of gal-3 in promoting cardiac fibrosis and subsequent heart damage, the inhibition of gal-3 has emerged as a potential antifibrotic therapeutic strategy. Preclinical studies evaluating modified citrus pectin (MCP), an oral gal-3 inhibitor, demonstrated promising results, including reduced inflammation, fibrosis, and angiogenesis in various animal models and in vitro studies [60]. Similar beneficial effects were observed in experimental models involving pressure overload or myocardial infarction, indicating possible protective properties against cardiac remodeling and dysfunction [61,62].

However, these positive outcomes have not yet been replicated in clinical trials. The first randomized clinical study assessing MCP did not show a significant reduction in organ fibrosis, although it confirmed the safety and good tolerability of the compound. These findings indicate that achieving a clinically meaningful antifibrotic effect through gal-3 inhibition might require longer treatment durations or possibly combination therapy [58].

Belapectin (GR-MD-02), a galactoarabino-rhamnogalacturonan derived from pectin, is among the most clinically advanced galectin-3 inhibitors. In a phase II clinical trial involving patients with liver fibrosis secondary to NASH, belapectin modestly reduced the incidence of new esophageal varices and slightly lowered portal vein pressure; however, its antifibrotic effect on liver tissue itself remained uncertain [63]. Another galectin-3 inhibitor, GB1107—a small-molecule oral inhibitor targeting the CRD domain—is currently under investigation. A phase I clinical trial (GB1211, ClinicalTrials.gov Identifier: NCT05009680) assessing safety and tolerability in patients with cirrhosis of various etiologies is ongoing, and definitive conclusions about efficacy cannot yet be drawn [64].

In summary, despite promising preclinical data, the clinical utility of galectin-3 inhibitors remains uncertain. Results from ongoing clinical trials will be essential to better define their therapeutic potential.

### 3.6. Galectin-3 and Myocardial Extracellular Volume

Myocardial extracellular volume (ECV), most commonly measured using magnetic resonance imaging (MRI) with T1 mapping, is an indicator of the degree of fibrosis and remodeling in the heart myocardium. Since gal-3 promotes fibrosis, the relationship between gal-3 concentrations and ECV is of particular interest. Several studies have demonstrated a significant positive correlation between blood concentrations of gal-3 and myocardial ECV.

In patients with a history of myocardial infarction, this relationship was demonstrated by Perea et al. [65]. A study involving 26 patients after STEMI measured biomarkers (gal-3, BNP) and performed MRI during the acute phase, with follow-up MRI at 6 months to assess ECV in the post-infarction myocardium. Gal-3 concentrations on day 7 post-infarction were found to be significantly and positively correlated with ECV values at 6 months (*r* = 0.428; *p* < 0.05). By comparison, BNP showed only a trend toward correlation (*r* = 0.38; *p* = 0.059). Moreover, gal-3 was an independent predictor of ECV increase in multivariate analysis; together with BNP, it explained about 30% of ECV variability (*R*^2^ = 0.34; *p* < 0.05). As mentioned earlier, exceeding a gal-3 threshold of > 10.15 ng/mL in the acute phase identified patients with significant ECV expansion with high sensitivity (*AUC* = 0.76). These findings suggest that circulating gal-3 during the peri-infarction period reflects an intensified fibrotic and remodeling process that contributes to increased extracellular matrix in the post-infarction healing phase. Gal-3 may serve as an early marker to identify patients prone to maladaptive cardiac scarring.

In chronic conditions such as heart failure with preserved ejection fraction (HFpEF), the relationship between gal-3 and ECV is also of interest. HFpEF is characterized by diffuse myocardial fibrosis, leading to impaired relaxation. Serial measurements of gal-3 in patients with HFpEF increased alongside fibrosis progression and were associated with worsening diastolic function, as inferred from E/e’ on echocardiography [66,67]. Further studies are needed that integrate precise imaging measurements (such as ECV from MRI) with biomarkers to more fully define the relationship between gal-3 and fibrotic burden in the heart among patients with chronic diseases. In summary, there is a consensus that gal-3 correlates with the degree of myocardial fibrosis as measured by ECV. In acute conditions (infarction, inflammation), elevated gal-3 predicts greater fibrosis during the healing phase [65,68]. In chronic diseases, gal-3 also reflects, at least partially, the fibrotic burden, although its concentration is influenced by multiple factors (e.g., kidney function).

Studies using ECV as the “gold standard” have shown that gal-3 is a useful, though nonspecific, marker of the extracellular matrix. ECV and gal-3 do not compete but rather complement each other: ECV provides direct information about cardiac fibrosis, while gal-3 offers a global view of pro-fibrotic and inflammatory activation in the body. In clinical practice, measuring gal-3 is incomparably simpler and cheaper than ECV imaging, making it suitable as a screening tool; in turn, elevated gal-3 may serve as an indication for advanced imaging to assess the extent of fibrosis.

### 3.7. Genetic Determinants of Circulating Galectin-3 Concentrations and Ethnic Differences

In recent years, genome-wide association studies have identified genetic variants significantly associated with serum gal-3 concentrations. A key breakthrough has been the identification of two major loci influencing gal-3: variants within the *LGALS3* gene (polymorphisms rs4644, rs4652) and the *ABO* gene, which determine blood groups. Variants in *LGALS3* may directly modulate gal-3 expression, a finding confirmed at both the transcriptomic and protein levels. Together, these variants explain up to 29% of the variability in serum gal-3 concentrations observed within the European population [69].

Regarding cardiovascular diseases, special attention has been paid to the potential links between gal-3-associated polymorphisms and risks of HF and atherosclerosis. Mendelian randomization studies, however, suggest that although elevated gal-3 concentrations serve as a marker of poor prognosis in CVDs, their causal role in the development of HF and vascular diseases remains uncertain [70,71].

In a large-scale proteogenomic study [72], using cis-Mendelian randomization analysis, higher gal-3 concentrations positively correlated with an increased risk of HF. Nonetheless, other Mendelian randomization analyses failed to confirm a direct causal link between genetically elevated gal-3 concentrations and HF risk. These findings imply that gal-3 is likely a biomarker reflecting cardiac fibrosis and remodeling rather than an etiological factor.

Importantly, ethnic differences might also influence gal-3 concentrations. A study conducted in an Asian population identified specific regulatory variants within *LGALS3*, differing in frequency compared to European populations. Notably, variant rs28379289, common in East Asian populations, was associated with increased *LGALS3* expression in placental tissues, indicating ethnic variation in gal-3 regulation [73].

**Figure 2:**
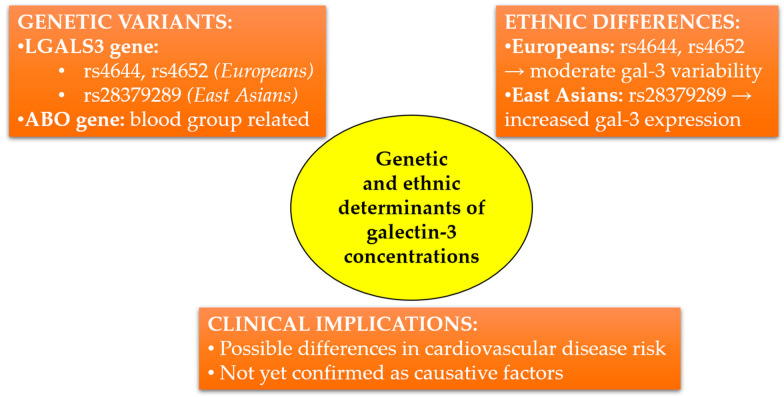
Genetic and ethnic determinants influencing circulating galectin-3 concentrations. *LGALS3* and *ABO* gene variants differ in frequency between European and East Asian populations, potentially affecting cardiovascular risk. Clinical implications remain to be fully elucidated.

From an epidemiological perspective, higher gal-3 concentrations observed among individuals of Asian descent might partially explain differences in cardiovascular disease incidence across populations. However, further studies incorporating both genetic and environmental contexts are required to precisely elucidate the clinical significance of these observations. The simplified diagram presented in Figure 2 summarizes the current knowledge on genetic variants influencing gal-3 concentrations, highlights ethnic differences, and outlines potential clinical implications.

## 4. Association of Galectin-3 with Individual Components of LS7 and LE8

### 4.1. Tobacco Smoking

The relationship between tobacco smoking and gal-3 concentrations has been analyzed in numerous studies. Substances contained in cigarette smoke may regulate gal-3 expression and activate related inflammatory and pro-fibrotic mechanisms. However, findings from both clinical and basic research are heterogeneous, indicating the complexity of this association and the need for cautious interpretation of the available data.

The study by de Boer et al. [74], which included a large population (*n* = 7968), showed a weak but significant negative correlation between tobacco smoking and gal-3 concentrations (*β* = −0.051, *p* < 0.001). However, after adjusting for sex, age, and kidney function, this association was no longer significant (*β* = 0.002, *p* > 0.05). Smoking may play a limited role as an independent determinant of gal-3 concentration, highlighting the importance of other factors such as renal function or BMI. Nevertheless, the authors did not analyze molecular mechanisms or the effect of smoking cessation on gal-3 concentrations.

Evidence pointing to a direct link between tobacco smoke and the activation of gal-3-related mechanisms was provided by the study by Sharma et al. [75], which observed increased gal-3 expression in AECs exposed to cigarette smoke extract (CSE). Gal-3 mRNA concentrations rose significantly (up to fivefold at 100 µM CSE, *p* < 0.001) and were associated with enhanced epithelial-to-mesenchymal transition. A key mechanism involved oxidative stress activating the RUNX-2/gal-3 axis. An antioxidant (N-acetylcysteine) and the gal-3 inhibitor GB1107 effectively suppressed these changes, suggesting potential therapeutic strategies.

Similar findings were presented in another study [76], which showed that nicotine directly increases gal-3 expression in breast cancer cells (MCF-7). This expression was dependent on activation of the α9 nicotinic acetylcholine receptor and the STAT3 pathway. Gal-3 expression was significantly higher in breast cancer tissues of smoking patients (twofold increase, *p* < 0.001). Additionally, gal-3 exerted a protective effect against mitochondrial apoptosis induced by cytostatic agents, indicating a role for nicotine and gal-3 in chemoresistance.

Clinically relevant insights are provided by the study [77], which assessed gal-3 concentrations in patients with chronic obstructive pulmonary disease (COPD). The comparative group consisted of never-smokers and former smokers. Gal-3 concentrations were significantly highest among current smokers, both during exacerbation and recovery phases. Additionally, gal-3 correlated with inflammatory markers such as hsCRP (*r* = 0.35, *p* < 0.05) and NT-proBNP (*r* = 0.32, *p* < 0.05). Smoking status was the only significant predictor of gal-3 concentrations (*β* = 0.458, *p* < 0.05). These findings indicate that gal-3 serves as a smoking-induced inflammatory biomarker, particularly relevant in patients with COPD.

A significant role of gal-3 in the response of EPCs to cigarette smoke was also described in the study [78]. Exposure of the cells to CSE resulted in a marked increase in gal-3 expression (6.7-fold mRNA increase at 8% CSE, *p* < 0.001) and induction of autophagy. The use of galectin-3 short hairpin RNA (a molecular biology technique used to silence the gene encoding gal-3) inhibited smoke-induced processes such as autophagy and endothelial cell dysfunction. This suggests a key role of gal-3 in the harmful cardiovascular effects of smoking.

Similarly, Zhang et al. [79] demonstrated that gal-3 was essential for the activation of the MUC1-C/EGFR pathway in epithelial cells exposed to cigarette smoke. Smoke exposure led to a threefold increase in gal-3 bound to MUC1-C (*p* < 0.05). This triggered activation of the pro-inflammatory Src/Jnk pathway, leading to E-cadherin degradation and epithelial-to-mesenchymal transition. Inhibition of MUC1 glycosylation prevented its interaction with gal-3 and suppressed the pathological effects, indicating the potential relevance of this mechanism as a therapeutic target.

The harmful effects of tobacco smoke, confirming the role of gal-3 as a mediator of oxidative stress and inflammation, were demonstrated by Nemmar et al. [80] in a vascular model of exposure to waterpipe smoke. Exposure led to an increase in gal-3 concentrations (*p* < 0.001) as well as inflammatory and oxidative stress markers (TNF-α, IL-1β, lipid peroxidation). These changes were associated with endothelial dysfunction and DNA damage, indicating a potential mechanism by which smoking affects the cardiovascular system.

Experimental data indicate that galectin-3 plays a significant role in the inflammatory response and oxidative stress induced by nicotine. In a study [81] conducted on rats, daily nicotine exposure (0.5 mg/kg/day) led to a significant increase in galectin-3 concentrations in lung tissue relative to the control group (*p* < 0.001). This was accompanied by elevated concentrations of pro-inflammatory cytokines: IL-6 and IL-1β (both: *p* < 0.05), as well as oxidative stress markers—malondialdehyde levels were significantly higher (*p* < 0.05), while the activity of antioxidant enzymes (GSH-Px, SOD) was reduced. Administration of dexpanthenol significantly lowered gal-3 concentrations and inflammatory markers, restoring them to values close to the control group, suggesting its potential protective effect. Notably, the study did not evaluate the effect of smoking cessation on gal-3 concentrations, which represents a significant limitation in the context of cardiovascular prevention and the reversibility of these changes.

Table 4 presents a summary of studies analyzing the effects of tobacco use (in various forms: cigarette smoke, nicotine, waterpipe smoke) on gal-3 concentrations, the molecular mechanisms regulating its expression, and associated biological effects.

In summary, the data indicate a significant, though not fully consistent, association between tobacco smoking and gal-3 concentrations, which may serve as a biomarker of inflammatory responses and oxidative damage induced by tobacco smoke components. However, the mechanisms regulating gal-3 expression require further investigation, particularly regarding the effects of smoking cessation, which have not been adequately addressed in existing studies.

### 4.2. Diet

Galectin-3 can be significantly modulated by dietary habits. In the LS7 scale, diet is one of the modifiable cardiovascular risk factors, potentially influencing the concentrations of gal-3.

Studies concluded that a high-fat diet (HFD) significantly increases gal-3 expression and activates inflammatory and fibrogenic pathways. It has been shown that consumption of a HFD induces gal-3 expression and activation of the NLRP3 inflammasome via TLR4 [83]. This heightens inflammatory processes in the liver and potentially offers a role in the pathogenesis of non-alcoholic steatohepatitis (NASH). Similar results were presented by Iacobini et al. [84], who, in a knockout mouse model (Lgals3−/−), demonstrated that the lack of gal-3 protected animals from diet-induced NASH. These findings suggest that limiting saturated fat intake may reduce the risk of chronic inflammatory and fibrotic processes mediated by gal-3.

Marín-Royo et al. [85] confirmed that excess saturated fats in the diet induce gal-3 overexpression in the hearts of rats, leading to lipotoxicity, oxidative stress, and mitochondrial dysfunction. The use of MCP, a gal-3 inhibitor, significantly reduced these adverse effects. This suggests potential benefits of diets rich in components that limit gal-3 activity.

The study by Gonçalves et al. [86] in rats fed a hypercaloric diet (rich in fats, simple sugars, and salt) also confirms that even short-term consumption of such a diet significantly increases gal-3 concentrations, resulting in cardiac hypertrophy and myocardial fibrosis. This evidence highlights the necessity of a healthy diet in the prevention of CVDs.

On the other hand, the absence of gal-3 may paradoxically exacerbate the metabolic consequences of a HFD, including increased adipose tissue mass and heightened inflammation [87]. This highlights the important role of gal-3 in maintaining metabolic balance in the context of various dietary habits. A healthy diet, by reducing pro-inflammatory factors associated with HFD, may support the protective function of gal-3 against the metabolic consequences of obesity and insulin resistance.

Wensvoort [88] presents a hypothesis that a Western diet rich in processed red meat may promote vascular remodeling through excessive activation of the elastin receptor complex (ERC) by elastin-derived peptides. Although the author did not conduct direct studies on gal-3, he included it in the model as a potential additional ligand of ERC, suggesting an indirect link to inflammation and vascular remodeling characteristic of metabolic syndrome. In conclusion, he recommends avoiding processed meats and other components that enhance chronic inflammation. A healthy diet should avoid pro-inflammatory components, potentially modulating mechanisms indirectly related to gal-3.

Important data on dietary interventions also come from studies on intermittent fasting (IF). A WONDERFUL trial (Weekly ONe-Day WatER-only Fasting InterventionaL trial for low-density lipoprotein cholesterol reduction) presented a significant increase in gal-3 concentrations after 26 weeks of IF [89]. This increase correlated with improvements in metabolic parameters, such as reductions in HOMA-IR, glucose, and insulin levels. Conversely, Lee et al. [90] and Kim et al. [91] demonstrated that IF effectively lowers gal-3 concentrations in mice fed a HFD, alleviating liver fibrosis, inflammation, and adipose tissue inflammation. This suggests that IF may modulate gal-3 concentrations, reducing the metabolic and inflammatory effects of excessive caloric and fat intake.

The result of dietary sodium content on kidney function and fluid–electrolyte balance has also been described in a mouse model with gal-3 deficiency [92]. Although the study did not directly address healthy dietary patterns, its findings suggest that sodium intake control may indirectly influence gal-3-regulated mechanisms, particularly those related to electrolyte balance and BP regulation.

Table 5 presents selected studies on the impact of dietary interventions on gal-3 concentrations and associated inflammatory and metabolic mechanisms. The results indicate that a HFD consistently leads to increased gal-3 concentrations and enhanced inflammatory responses, while intermittent fasting may modulate gal-3 concentrations depending on the physiological context.

In summary, the available data indicate that a diet rich in saturated fats, simple sugars, and processed red meat significantly increases gal-3 concentrations, exacerbating inflammation, fibrosis, and metabolic disturbances. In contrast, dietary interventions such as IF or diets that limit saturated fat intake can effectively modulate gal-3 concentrations, contributing to improvements in metabolic parameters, reduction of inflammation, and potential cardiovascular protection. However, data are still lacking regarding the effects of healthy dietary components (e.g., antioxidants, PUFAs, fiber) on gal-3 regulation, which represents an important direction for future research.

### 4.3. Physical Activity

Gal-3 concentrations can be modulated by both acute, regular physical activity and intense physical exertion. Physical activity is a component of both the LS7 and LE8 scales.

It has been shown that acute high-intensity interval training (HIIT) leads to a significant increase in serum gal-3 concentrations (by an average of 39.5%, *p* < 0.001), in both runners and cyclists [93]. The authors associated these changes with endothelial activation. They suggested that short-term increases in gal-3 may reflect a response to mechanical vascular stress and the initiation of endothelial remodeling processes, potentially even fibrotic changes via endothelial-to-mesenchymal transition. However, the main limitations of this study were its short observation period and small sample size (*n* = 18), which make it difficult to assess the clinical significance of the findings.

Similar observations were made by Kaleta-Duss et al. [94] among 35 amateur marathon runners, demonstrating a transient increase in gal-3 after the marathon (from an average of 8.53 to 10.65 ng/mL, *p* < 0.05). Galectin-3 concentrations returned to baseline values two weeks after the run. The increase in gal-3 correlated with an increase in heart-type fatty acid binding protein (*r* = 0.52, *p* < 0.05), suggesting the possibility of adaptive changes related to cardiac load. Further evidence for the transient, adaptive nature of gal-3 increase is provided by findings from Vassalle et al. [95], who observed a significant but short-term increase in gal-3 (by an average of 33%, *p* < 0.001) in half-marathon runners, which normalized within 24 h. The same was shown in the study by Le Goff et al. [96], where the increase in gal-3 was significantly more pronounced in ultramarathon runners (from 9.39 ± 2.94 ng/mL to 19.8 ± 4.39 ng/mL, *p* < 0.001) than in runners of shorter distances (from 9.23 ± 2.56 ng/mL to 23.9 ± 5.59 ng/mL, *p* < 0.001). In the reference group, which ran 10 km, the increase in galectin-3 was less marked (from 10.8 ± 2.63 ng/mL to 14.1 ± 3.38 ng/mL). Three hours after exertion, gal-3 concentrations returned to near-baseline values in all groups, suggesting that the biomarker increase after intense exercise is transient. The study by Lewicka-Potocka et al. [97] complements these observations, indicating that intense endurance exercise (marathon) leads to an increase in gal-3 concentrations, which negatively correlate with right ventricular (RV) ejection fraction (*r* = −0.48, *p* < 0.05) and with reduced RV radial shortening. Elevated gal-3 concentrations were connected with lower VO_2_ max (*r* = −0.47, *p* < 0.05), suggesting that intense physical effort may induce adaptive RV remodeling associated with a transient elevation in this biomarker.

The issue of the source of gal-3 increase during exercise has also been analyzed in animal models of vascular and cognitive disorders. It has been shown that regular physical activity significantly reduces gal-3 expression in microglia and white matter [98,99], leading to a reduction in neuroinflammation and improvement in cognitive function in mice with vascular dementia. The evidence implies that a potential neuroprotective effect of regular physical exercise through gal-3 modulation, which may also have implications for CVH.

The beneficial results of regular physical activity are also confirmed by the study [100], which demonstrated that an eight-week training program (either combined or HIIT) significantly reduced gal-3, CRP, and fibrinogen concentrations in patients with CAD after COVID-19 (*p* < 0.05). Meanwhile, the randomized controlled trial by Keyhani et al. [101] indicates the potential for a significant reduction in gal-3 gene expression in postmenopausal women, both following HIIT (a reduction of 94.15%) and with moderate aerobic activity (84.74%), which was also associated with improvement in lipid profile.

In contrast to the studies described above, the findings of Ahmad et al. [102] suggest an association between elevated gal-3 concentrations and reduced physical performance in patients with HF, where elevated gal-3 concentrations were inversely correlated with the results of the short physical performance battery (*r*^2^ = 0.089, *p* < 0.05) and handgrip strength (*r*^2^ = 0.078, *p* < 0.05).

The study by Pacheco et al. [103] indicates that physical activity status in older adults may influence the expression of galectin-3 binding protein (LGALS3BP), although no significant differences in gal-3 concentrations were observed. The evidence implies that while physical activity may have a beneficial effect on the expression of related proteins regulating inflammation, the direct impact of exercise on gal-3 requires further investigation.

Salvagno et al. [104] demonstrated that extreme physical exertion (a 60 km ultramarathon) causes a transient increase in gal-3 concentrations (2.4-fold increase; *p* < 0.001), which, however, was not correlated with cardiac injury biomarkers (TnI and NT-proBNP). This suggests that gal-3 may reflect an adaptive response of the body to extreme exertion rather than actual myocardial damage.

Table 6 presents a synthetic summary of findings from experimental and clinical studies analyzing the impact of physical activity, both acute and chronic, on galectin-3 concentrations. The table allows comparison of training effects depending on intensity, duration, and participants’ health status, and also points to potential mechanisms underlying changes in gal-3 concentrations.

In summary, intense, short-term physical activity leads to a transient increase in gal-3, which likely reflects adaptive changes within the cardiovascular system and endothelium. Regular exercise, particularly aerobic training or HIIT, tends to reduce gal-3 concentrations, suggesting a role in long-term cardiovascular and cognitive protection. However, the long-term effects of repeated gal-3 elevations in individuals engaged in high-volume endurance sports require further investigation. Larger prospective studies are also necessary to evaluate the clinical significance of gal-3 modulation through physical activity in reducing the risk of CVDs.

### 4.4. BMI

Gal-3 concentration may be significantly modulated by body mass and fat distribution, particularly abdominal obesity.

A study conducted in a large Chinese population demonstrated a significant, though moderate, association between gal-3 concentration and BMI (*R* = 0.07, *p* < 0.05). This correlation lost statistical significance after adjusting for gender and age (*β* = 0.04; 95% CI: −0.02, 0.1; *p* = 0.15) [108]. A stronger correlation was noted with waist circumference (WC), which remained significant even after multivariate regression analysis (*β* = 0.12; 95% CI: 0.04, 0.21; *p* < 0.05). This suggests that visceral obesity is a better predictor of gal-3 concentration than BMI alone. Gal-3 concentration also correlated with other metabolic markers, including triglycerides (*β* = 0.11; *p* < 0.001) and hs-CRP (*β* = 0.08; *p* > 0.05), which may indicate a role for this biomarker in obesity-related inflammation.

Similar associations were confirmed by Weigert et al. [109], who analyzed gal-3 concentrations in individuals with normal BMI, overweight, and type 2 diabetes mellitus (T2DM). They found significantly elevated gal-3 concentrations in those with overweight and T2DM compared to the control group. Gal-3 concentration showed a positive association with BMI (*r* = 0.357; *p* < 0.05). Notably, the study also observed higher gal-3 concentrations in visceral compared to subcutaneous adipose tissue, suggesting a key role of visceral fat as a source of this biomarker’s synthesis.

Important data on the association between gal-3 and obesity were also provided by the study of Suthahar et al. [110], conducted in the large PREVEND cohort (8202 participants aged 28–75 years, with a median follow-up of 11.3 ± 3.1 years). It was shown that gal-3 concentration significantly increased with rising BMI (obesity: 11.7 ± 2.3 vs. normal body weight: 10.2 ± 1.9 ng/mL, *p* < 0.001). However, unlike other biomarkers such as NT-proBNP or cTnT, gal-3 did not demonstrate significant prognostic value for HF risk in the context of BMI.

The study [111], conducted in a population of 8687 individuals within the ARIC project (Atherosclerosis Risk in Communities Study), provides important clinical evidence on the relationship between high BMI, increased gal-3 concentrations, and HF risk. Individuals with severe obesity (BMI ≥ 35 kg/m²) had more than twice the risk of elevated gal-3 concentration (*OR* = 2.32; *p* < 0.001). This relationship was notably strong in the subgroup of women with severe obesity (*OR* = 3.00), suggesting potential gender differences. Moreover, the coexistence of severe obesity and high gal-3 was connected with more than a fourfold increased risk of HF (*HR* = 4.19; *p* < 0.001).

Interesting findings regarding the impact of weight reduction on gal-3 concentrations were presented by Aksit et al. [112], who assessed patients with obesity undergoing bariatric surgery. Initially, gal-3 concentrations were significantly higher in the group with class III obesity (17.6 ± 4.2 vs. 14.1 ± 3.0 ng/mL; *p* < 0.05) and presented a positive correlation with BMI (*r* = 0.375; *p* < 0.001). After surgery, although a downward trend in gal-3 concentration was observed at 3 and 6 months, these changes did not reach statistical significance, suggesting that the observation period was too short to capture meaningful changes in this biomarker.

Fryk et al. [113], analyzing a population from the POEM study (Prospective Investigation of Obesity, Energy and Metabolism, *n* = 502), reported different findings. In their analysis, gal-3 did not show significant correlations with BMI (*β* = 0.07; *p* = 0.095) or with indicators of fat distribution. These findings indicate that gal-3 might have a minor role in adipogenesis regulation itself or may act as a marker of inflammation originating from sources other than adipose tissue.

Table 7 presents a synthetic summary of findings from studies evaluating the association between gal-3 concentration and BMI as well as obesity-related parameters (including visceral fat and WC).

In summary, available data suggest a significant, though variable, association between gal-3 and BMI, with abdominal obesity appearing to be more strongly linked to this biomarker’s concentration than BMI alone. Gal-3 is also associated with inflammatory markers, which may indicate its involvement in the pathophysiology of chronic inflammation accompanying obesity. Further research should focus on elucidating the molecular mechanisms connecting gal-3 with different types of obesity and on evaluating the potential for modulating gal-3 concentration through weight-reduction interventions.

### 4.5. Cholesterol

The association between gal-3 and lipid metabolism, as well as its potential impact on cholesterol regulation, is the subject of growing interest, particularly in the context of CVDs. This subsection reviews current evidence on the relationship between lipid profile and gal-3 (with particular focus on HDL, LDL, and total cholesterol).

Zeng et al. [114] demonstrated that a high gal-3 concentration combined with low HDL-C significantly strengthens the risk of death and cardiovascular events within one year after ischemic stroke. Notably, despite the lack of a statistical correlation between HDL-C and gal-3 concentrations (*r* = −0.005, *p* = 0.776), their synergistic impact on prognosis was evident; the *HR* for vascular events was 1.92 (95% CI: 1.26–2.94). These findings suggest that gal-3 may function not only as a marker but could also be actively involved in the pathogenesis of atherosclerotic processes, particularly in the presence of lipid abnormalities.

At the experimental level, Tan et al. [115] demonstrated that inhibition of gal-3 expression in a rat model of atherosclerosis resulted in an improved lipid profile (decrease in TG, LDL-C, TC, and elevation in HDL-C), suggesting a potential role of gal-3 in cholesterol metabolism. This mechanism appears to be mediated by the regulation of cholesterol ester hydrolase expression, indicating a possible pathogenic role of gal-3 in lipid accumulation within the vascular wall. A limitation of the study is its animal model nature, which restricts the direct applicability of the results to humans.

In the clinical study by Winter et al. [116] involving participants with premature myocardial infarction, a positive correlation was obtained between gal-3 concentration and non-HDL cholesterol (*r* = 0.23, *p* < 0.05) as well as remnant cholesterol (*r* = 0.21, *p* < 0.05). This evidence is consistent with the hypothesis that gal-3 may be involved in the pathogenesis of atherogenic dyslipidemia, although no direct correlation was observed with TC or LDL-C. Importantly, in gal-3 knockout animal models, reduced atherosclerotic lesions were observed under a high-fat diet, further supporting its role in atherogenesis.

Analysis of data from the PREVEND cohort [3] indicated that total cholesterol was an independent factor associated with gal-3 concentration in cross-sectional analysis, but it did not significantly affect its variability over time. This may suggest that TC concentration influences the baseline gal-3 concentration but does not determine its dynamic changes, which could be relevant for planning strategies to monitor this biomarker.

It is also worth highlighting the data from Melin et al. [117], who have shown a positive correlation between gal-3 binding protein and TC concentration in patients with type 1 diabetes, particularly in the male subgroup (*AOR* = 2.3; *p* < 0.05). This may suggest a link between gal-3 and an unfavorable lipid profile even in younger, metabolically burdened populations.

In summary, the data suggest that galectin-3 may act not only as an inflammatory marker but also as an active participant in the pathophysiology of atherosclerosis, indirectly influencing lipid metabolism—particularly in the context of low HDL-C and elevated non-HDL cholesterol. However, most studies are correlational in nature (cohort studies, animal models), with a limited number of prospective or randomized trials. To date, no definitive evidence has confirmed a direct role of gal-3 on TC concentration, which represents a significant limitation of current knowledge. Translational and interventional research is necessary to evaluate the impact of gal-3 modulation on lipid parameters and long-term cardiovascular risk.

### 4.6. Blood Pressure

Gal-3 is a key regulator of vascular homeostasis, and its activity involves both inflammatory processes and vascular wall remodeling. Although a direct association between galectin-3 concentration and BP measurements has not been clearly confirmed in clinical studies, numerous experimental data imply that gal-3 may play a role in the pathogenesis of AH through its impact on vascular stiffness, vascular remodeling, and endothelial dysfunction.

Pioneering studies by Nangia-Makker et al. [118] provided some of the first evidence that gal-3 exhibits activity modulating endothelial function and angiogenesis. In both in vivo and in vitro models, it was shown that gal-3 induces morphogenesis of endothelial cells (HUVEC) and the formation of microvessels in Matrigel matrix. Although this mechanism does not directly relate to BP values, it provides key biological foundations for understanding the impact of gal-3 on the microcirculation and potential changes in vascular tone. From the perspective of current data, the angiogenic activity of gal-3 may be relevant to the development of microangiopathy in the course of AH, though this requires further translational research.

In one research paper [119] using a mouse model with angiotensin II infusion, it was demonstrated that gal-3 expression in the endothelium significantly increases under the influence of angiotensin II, a change not observed in gal-3 knockout mice (gal-3−/−). Moreover, the absence of gal-3 protected the animals from an increase in systolic BP and endothelial dysfunction, as well as reduced ROS production, NOX2 expression, and inflammatory markers (IL-6, VCAM-1). These conclusions are based on experimental in vivo models and functional vascular analyses (e.g., response to ACh and SNP), which positions the strength of evidence as solid in the context of preclinical research.

Similar mechanisms were confirmed in the study [120], where in a model of induced hypertension, gal-3 was shown to be involved in the activation of the AMPK/TXNIP and PI3K/Akt pathways, resulting in enhanced vascular fibrosis and vascular wall remodeling. The authors also emphasize that gal-3 affects endothelial homeostasis through interactions with the extracellular matrix and modulation of the inflammatory response. Although the study does not provide numerical data on BP values, the described mechanisms are potentially significant in the context of vascular stiffness—a key component of AH pathophysiology.

Further confirmation of the role of gal-3 as an indicator of endothelial dysfunction comes from the study by Hsu et al. [121], involving individuals with chronic kidney disease (CKD). An inverse correlation was described between gal-3 concentration and the vascular reactivity index (*r* = −0.439, *p* < 0.001), independent of eGFR and inflammatory markers. These findings support the concept of gal-3 as a marker of endothelial injury, which over time may lead to AH.

In a study involving patients with AH, gal-3 concentration showed a positive correlation with systolic BP values (*r* = 0.225, *p* < 0.05); however, this association was not significant in a multivariate model. A much stronger relationship was observed between gal-3 and indicators of left ventricular remodeling, such as left ventricular mass and index, suggesting the potential usefulness of this biomarker in assessing hypertension-related complications [122]. The authors highlight the higher predictive value of gal-3 compared to BNP (*AUC*: 0.698 vs. 0.53), emphasizing the diagnostic potential of this molecule, though not necessarily as a direct predictor of BP.

Data from the reviews indicate that gal-3 is involved in modulating the characteristics of vascular smooth muscle cells, facilitating their osteogenic transformation and type I collagen synthesis, which leads to medial layer thickening and increased vascular stiffness [123,124]. Although these findings are molecular and review-based, they are consistent with experimental observations and support the hypothesis of a functional role of gal-3 in the pathogenesis of AH.

Clinical studies focusing specifically on the relationship between gal-3 concentration and BP values are scarce and yield inconclusive results. For instance, in the study [125], involving patients with AH and HF, no differences in gal-3 concentration were described between groups, and its relationship with BP values was not assessed. The absence of numerical data and regression analysis prevents drawing conclusions about the importance of gal-3 as a marker of AH in this population.

Table 8 presents a summary of selected molecular and cellular mechanisms involving gal-3 and their associations with key pathophysiological phenomena leading to increased vascular stiffness, endothelial dysfunction, and elevated vascular resistance.

In summary, gal-3 may play a key role in the AH pathogenesis by enhancing inflammatory processes, fibrosis, and vascular remodeling (including its impact on endothelial function). However, clinical evidence regarding its direct association with BP values remains inconclusive. Current knowledge suggests that gal-3 acts more as an indirect mediator of vascular injury and endothelial dysfunction rather than as a biomarker of BP itself.

### 4.7. Glucose Concentration

Gal-3 is increasingly being applied in research on the pathophysiology of T2DM and its cardiovascular complications. Despite a growing number of reports, the relationship between glucose concentration (both fasting blood glucose, FBG, and glycated hemoglobin, HbA1c) and gal-3 concentration remains inconclusive, with study findings suggesting a stronger association with the chronic effects of hyperglycemia rather than with glucose concentration itself.

In the published review [126], significant expression of gal-3 in the heart and its increase under metabolic conditions such as diabetes were highlighted. The authors emphasize its potential role in diabetic cardiomyopathy (DCM); however, the lack of quantitative data prevents an assessment of the strength of the relationship between glucose concentration and gal-3. In another study [127], despite no differences in HbA1c between patients with and without HFpEF, higher baseline gal-3 concentration was an independent predictor of HF development (*AUC* = 0.876; *p* < 0.001). It suggests that gal-3 may reflect fibrosis and inflammatory processes independent of glycemia.

The study by Flores-Ramírez et al. [128] demonstrated significantly higher gal-3 concentration in individuals with T2DM, regardless of left ventricular ejection fraction (LVEF). The observations suggest an association between gal-3 and subclinical cardiac dysfunction. Jin et al. [129] described a significant association between gal-3 and HbA1c (*r* = 0.217; *p* < 0.05), with no significant associations with FBG or HOMA-IR. This indicates that gal-3 may be more related to chronic rather than acute dysregulation of glycemia.

In the study by Tan et al. [130], a weak but significant correlation between HbA1c and gal-3 (*r* = 0.06; *p* < 0.05) was demonstrated, with no association with T2DM duration or BMI. Gal-3 was described as an independent prognostic factor for mortality risk (*HR* = 1.17; 95% CI: 1.01–1.35; *p* < 0.05). These observations support its role as an indicator of a chronic inflammatory-fibrotic process, independent of classical metabolic markers.

Data from Kuzan et al. [131] confirmed significant correlations of gal-3 with FBG (*r* = 0.39; *p* < 0.001) and HbA1c (*r* = 0.267; *p* < 0.05) in a geriatric population, as well as the impact of metformin treatment on lowering gal-3 concentration (*p* < 0.05). This suggests the possibility of hypoglycemic pharmacotherapy influencing the expression of this protein, which may have therapeutic implications. Similar findings were reported by Weigert et al. [109], who demonstrated a negative correlation between HbA1c and gal-3 (*r* = −0.323; *p* < 0.05) and a metformin-induced reduction of gal-3 expression in vitro.

A comprehensive analysis was conducted by Vora et al. [132], documenting a relationship between baseline gal-3 concentration and the development of T2DM over long-term follow-up (*OR* = 1.20; 95% CI: 1.00–1.45; *p* < 0.05). Importantly, this relationship was independent of BMI and not associated with HOMA-IR, while gal-3 correlated with C-peptide and inflammatory markers. This suggests that gal-3 may serve as a marker of β-cell injury or dysfunction rather than insulin resistance alone.

Strong correlations of gal-3 with OGTT, FPG, and HOMA-IR parameters were demonstrated by Yilmaz et al. [133]. The diagnostic value of gal-3 was high (*AUC* = 0.912 for diabetes; *AUC* = 0.901 for prediabetes), indicating the potential application of this protein as a marker for early detection of glycemic disorders.

Important experimental data are also provided by Fantauzzi et al. [134], in which mice with gal-3 gene deletion exhibited impaired glucose tolerance, insulin resistance, and β-cell dysfunction. These observations support the hypothesis that gal-3 functions not only as a marker but also as an active participant in metabolic processes, including the regulation of insulin sensitivity and insulin secretion.

Table 9 presents a synthetic summary of clinical and experimental studies analyzing the association between gal-3 concentration and carbohydrate metabolism results such as fasting glycemia, HbA1c, insulin resistance, or the risk of developing T2DM.

In summary, available data indicate that gal-3 is associated with T2DM and its complications. However, the relationship with glucose concentration and HbA1c is inconsistent—some studies report correlations with HbA1c [109,131,135], while others do not confirm significant associations with glycemia [127,128]. The role of gal-3 as a mediator of pathogenic processes is supported by both clinical and experimental studies; however, further prospective analyses are needed to assess its diagnostic and prognostic utility in the context of glucose metabolism disorders.

### 4.8. Sleep Health

Gal-3 is a biomarker associated with sleep disorders, particularly in terms of sleep quality, inflammation, and cardiovascular consequences related to obstructive sleep apnea (OSA). The sleep aspect is included in the LE8 metric, but not in the older LS7.

In a cross-sectional population-based research paper [108], it was demonstrated that impaired sleep quality was significantly associated with elevated gal-3 concentration, with a significant *OR* of 1.68 (95% CI: 1.05–2.68) in a fully adjusted model that accounted for factors such as age, gender, smoking, alcohol consumption, AH, BMI, WC, FBG, TG, HDL, and hs-CRP. In contrast, nighttime sleep duration (<7 h vs. ≥7 h) and daytime nap duration (≥60 min vs. <60 min) did not show significant correlation with gal-3 concentration, suggesting that sleep quality, rather than sleep duration, may be crucial for this biomarker. In this study, the mechanisms were linked to activation of the hypothalamic–pituitary–adrenal axis, increased sympathetic nervous system activity, and elevated production of pro-inflammatory cytokines (IL-6, TNF-α). Additionally, the authors noted limitations including the lack of objective sleep assessment methods (e.g., polysomnography) and the cross-sectional design, which prevents definitive causal conclusions.

The relationship between gal-3 and OSA was also thoroughly discussed by Andersen et al. [136], who emphasized the role of this biomarker in inflammatory processes and fibrosis, particularly in the context of sleep fragmentation in patients with OSA. Patients with OSA were found to have substantially elevated gal-3 levels, which correlate with both disease severity and atherosclerotic burden in blood vessels. The authors suggest that gal-3 may influence neuroinflammatory mechanisms related to sleep disorders through microglial activation, involving corticotropin-releasing hormone and autophagy dysregulation. From a clinical standpoint, a key observation is that treatment with CPAP reduced gal-3 concentration, indicating the potential for therapeutic intervention to modulate this biomarker and decrease inflammation.

Clinical studies confirm a strong relationship between OSA severity and gal-3 concentration. Hamdi Pusuroglu et al. [137] found that mean gal-3 concentration significantly increased with the severity of OSA (mild: 4.3 ± 1200 ng/mL, moderate: 5.1 ± 1.4 ng/mL, severe: 6.4 ± 1700 ng/mL; *p* < 0.001). In addition, gal-3 concentration positively correlated with the apnea–hypopnea index (*r* = 0.337; *p* < 0.001) and the number of atherosclerotic plaques in coronary arteries (*r* = 0.417; *p* < 0.001). In multivariate analysis, gal-3 was an independent predictor of OSA severity (*OR* = 2.329; 95% CI: 1.558–3.480) and coronary artery atherosclerosis (*OR* = 2.175; 95% CI: 1.425–3.320). The authors associate this with increased inflammation, hypoxia, and oxidative stress, highlighting the potential of gal-3 as a cardiovascular risk biomarker in individuals with OSA.

Singh et al. [138] expand on these observations by highlighting sex differences in the correlation between gal-3 and OSA. Median gal-3 concentrations were significantly higher in severe and moderate OSA (7.63 ng/mL, *n* = 471) compared to individuals without or with mild OSA (6.53 ng/mL; *p* < 0.001). Multivariate analysis showed a significant association, particularly in women (*β* = 0.14; *p* < 0.001), whereas in men, these results were not significant (*p* = 0.86). The mechanism underlying these sex-specific differences remains unclear, although differences in inflammatory responses, microcirculatory function, and the effects of hypoxia and hypothalamic–pituitary–adrenal axis activation have been suggested. However, the authors note limitations due to the cross-sectional design of the study and the relatively small number of men in the analyzed population.

Khalaji et al. [139] and Zong et al. [140] complement these findings by providing context on the neurocognitive consequences of OSA related to gal-3 concentration. The studies suggest that elevated gal-3 concentration in individuals with severe OSA reflects intensified neuroinflammatory processes, oxidative stress, and reduced cognitive function (significantly lower Montreal Cognitive Assessment scores: 22.41 ± 2.56 vs. 27.11 ± 1.32 in the reference group; *p* < 0.001). After CPAP therapy, a significant decrease in gal-3 concentration was observed (*p* < 0.001), along with improvements in sleep parameters and cognitive performance (24.86 ± 1.81 in the score; *p* < 0.05), confirming the role of sleep quality in the regulation of this biomarker.

Table 10 presents a synthetic summary of current literature data regarding the relationship between gal-3 and sleep quality parameters, the severity and presence of OSA, as well as neuroinflammatory and CVH consequences. Both original and review studies are included, indicating the possible application of gal-3 as a biomarker of chronic inflammation, fibrosis, and risk of complications in the context of sleep disorders. A major limitation of most studies is their cross-sectional design and the lack of objective sleep assessment methods, which highlights the need for further prospective research using techniques such as polysomnography.

In summary, data from available studies indicate that gal-3 is significantly associated with sleep quality, OSA severity, and the inflammatory and neurocognitive consequences of this disorder. However, due to methodological limitations, including the lack of objective sleep assessment tools and the cross-sectional design of the studies, further analyses using precise diagnostic methods are necessary to gain a more comprehensive understanding of this relationship.

## 5. Limitations and Future Directions

This review is narrative in nature, which involves limited standardization in the process of data selection and analysis. Although the structure follows the SANRA scale, subjectivity in the selection of literature and interpretation of results cannot be excluded. Some of the referenced data come from preclinical or observational studies, which limits the possibility of direct translation into clinical practice. Many findings are based on correlations without clear confirmation of causal relationships. Additionally, despite promising data regarding gal-3 inhibitors, their use in clinical practice remains experimental.

Despite its promising role, gal-3 has several important limitations that restrict its clinical utility. Firstly, gal-3 lacks high specificity: elevated concentrations are not exclusive to HF or fibrosis-related pathologies but are also observed in chronic kidney disease, malignancies, and systemic inflammatory conditions. This non-specificity may confound clinical interpretation. Secondly, a significant inter-assay variability exists among commercially available gal-3 measurement kits, and there is no universally accepted standard for its quantification, which complicates comparisons between studies and clinical implementation. Moreover, even mild renal impairment independently elevates circulating gal-3 concentrations, thus potentially acting as a confounding factor when interpreting biomarker results in patients with concurrent cardiac and renal dysfunction. These limitations must be carefully considered when attempting to translate gal-3 measurement into routine clinical practice.

In the future, attention should be focused on:

1. Prospective studies evaluating the predictive value of gal-3 in various risk populations;
2. Interventional trials assessing the impact of lifestyle modifications on gal-3 concentration and related clinical outcomes;
3. Validation of gal-3 as a marker of therapeutic response in CVDs, particularly in HF and atrial fibrillation;
4. Assessment of its role as a marker of environmental and lifestyle effects in the context of LE8/LS7.

The interplay between lifestyle, occupational exposures, and emerging molecular biomarkers, including gal-3, warrants further translational research, as also emphasized in recent proposals for precision diagnostics in AH [1].

From a clinical perspective, in the near future, gal-3 may find application primarily in specialized cardiology clinics, e.g., in assessing the severity of HF with a mixed etiology (where a fibrotic component is suspected, as in HFpEF), or in patients with recurrent hospitalizations to evaluate their long-term prognosis. It may also be useful in the context of screening in at-risk populations, for instance, in patients with long-standing AH or T2DM, where elevated gal-3 concentration could indicate the onset of organ damage and prompt more aggressive risk factor control. However, before this becomes routine, it is necessary to establish clear management protocols for defined gal-3 concentrations (e.g., whether >17 ng/mL is considered high-risk, requiring therapy intensification).

In summary, gal-3 is a promising cardio-metabolic indicator that enhances our ability to assess patients beyond traditional risk factors. It serves as a link between lifestyle and risk factors (as captured in LS7/LE8) and actual organ damage; increased gal-3 concentration suggests that long-term exposure to risk factors has already translated into tangible pathological changes (fibrosis, remodeling). The implementation of gal-3 testing in daily practice should go hand-in-hand with staff education regarding result interpretation and the limitations of the test. Its clinical application may, in the future, facilitate the identification of patients who require more intensive preventive or therapeutic interventions. A vision of targeted therapy is also on the horizon. If the development of gal-3 inhibitors proves successful, we may one day be able not only to measure but also to modulate this parameter, slowing the progression of HF in our patients. As of today, gal-3 is an important piece of the puzzle, but it does not replace classical methods; it rather complements the diagnostic and prognostic profile of the patient. Although the path toward full clinical implementation of gal-3 is still ahead of us, this biomarker represents one of the most promising links between precision medicine and primary prevention of chronic diseases. Clinicians should stay updated with new guidelines and research findings, as the role of galectin-3 may evolve in the coming years as new evidence emerges.

## 6. Conclusions

**Figure 3:**
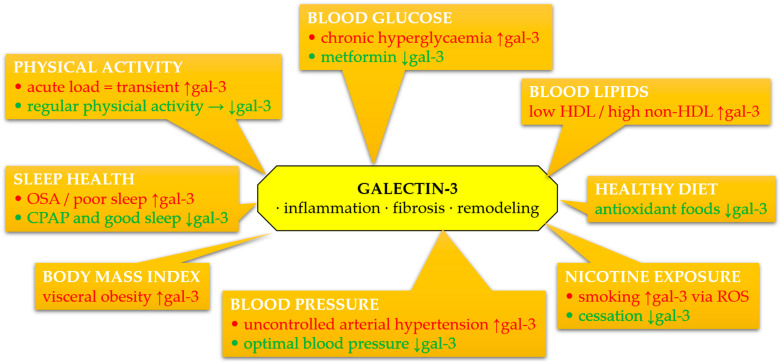
Schematic summary of associations between circulating galectin-3 (gal-3) and components of Life’s Essential 8 and Life’s Simple 7 metrics. Galectin-3 levels may be modulated by lifestyle and clinical factors, contributing to inflammation, fibrosis, oxidative stress, and extracellular matrix remodeling. Green color denotes favorable effects (decrease in gal-3), red color denotes adverse effects (increase in gal-3). Abbreviations: CPAP, continuous positive airway pressure; gal-3, galectin-3; HDL, high-density lipoprotein; OSA, obstructive sleep apnea; ROS, reactive oxygen species.

The complex interplay between gal-3 and individual components of Life’s Essential 8 metrics is summarized in Figure 3. This overview highlights potential mechanisms linking lifestyle, metabolic risk factors, and gal-3-driven cardiovascular pathology.

Galectin-3 is increasingly recognized as a pivotal mediator in the pathophysiology of cardiovascular diseases, particularly through its involvement in inflammation, fibrosis, and myocardial remodeling. Available data indicate that gal-3 is not only a marker of these processes but may also actively participate in their modulation. Its concentration correlates with various CVH parameters assessed within the LS7 and LE8 frameworks, although it is not directly included in these scales. The integration of gal-3 into the context of primary and secondary prevention requires further validation, but existing evidence suggests the possibility of using this molecule as a complementary biomarker of cardiovascular risk and response to lifestyle interventions.

## Figures and Tables

**Table 1:** The AHA Life’s Simple 7 scale for adults, based on Lloyd-Jones et al. [11].

	Goal	Health Goal Category	Definition
HEALTH BEHAVIORS	Current smoking	POOR	Current
		INTERMEDIATE	Former ≤ 12 months
		IDEAL	Never or quit > 12 months
	Healthy diet score	POOR	0–1 components ^a^
		INTERMEDIATE	2–3 components ^a^
		IDEAL	4–5 components ^a^
	Physical activity	POOR	Complete absence of weekly exercise
		INTERMEDIATE	Engaging in 1–149 min per week of moderate-intensity activity, or 1–74 min per week of vigorous-intensity exercise, or a combination of moderate and vigorous efforts not exceeding 149 min in total.
		IDEAL	A weekly total of ≥150 min of moderate-intensity physical activity, or ≥75 min of vigorous-intensity exercise, or an equivalent combination of both intensity levels
	Body mass index	POOR	≥30 kg/m^2^
		INTERMEDIATE	25–29.9 kg/m^2^
		IDEAL	<25 kg/m^2^
BIOLOGICAL METRICS	Total cholesterol	POOR	≥240 mg/dL
		INTERMEDIATE	200–239 mg/dL or under effective pharmacological control ^c^
		IDEAL	<200 mg/dL ^b^
	Blood pressure	POOR	SBP ≥ 140 mmHg or DBP ≥ 90 mmHg
		INTERMEDIATE	SBP 120–139 mmHg or DBP 80–89 mmHg or under effective pharmacological control ^d^
		IDEAL	<120/<80 mmHg ^b^
	Fasting plasma glucose	POOR	≥126 mg/dL
		INTERMEDIATE	100–125 mg/dL or treated to goal ^e^
		IDEAL	<100 mg/dL ^b^

Abbreviations: DBP, diastolic blood pressure; SBP, systolic blood pressure. ^a^ In accordance with the healthy diet score [12], the recommendations include the following components: (1) reducing daily sodium intake to below 1500 mg and (2) limiting caloric intake from beverages with added sugars to ≤450 kcal per week (approximately 36 ounces). (3) Regular intake of fiber-dense whole grains–defined as containing ≥1.1 g per 10 g of carbohydrates—is recommended at a minimum of three 1-ounce-equivalent servings every day. (4) The consumption of at least two servings (3.5 oz each) of fish per week, preferably oily fish, is also advised. Furthermore, (5) a daily intake of 4.5 cups or more of vegetables and fruits is encouraged to support optimal cardiometabolic health. ^b^ untreated values. ^c^ In accordance with guidelines [13]: For individuals at very high cardiovascular risk undergoing secondary prevention, clinical guidelines advise lowering LDL-C levels to <1.4 mmol/L (55 mg/dL) and achieving ≥50% reduction from baseline values. Similarly, in the context of primary prevention—regardless of the presence of familial hypercholesterolemia—a comparable therapeutic target is recommended, namely an LDL cholesterol concentration of <1.4 mmol/L along with a ≥50% decrease relative to the initial level. ^d^ In accordance with guidelines [14]: The initial therapeutic goal in arterial hypertension management should be to reduce blood pressure < 140/90 mmHg in all individuals. If this target is achieved without adverse effects, further lowering to values around or below 130/80 mmHg is recommended for the majority of patients. In adults under the age of 65 receiving antihypertensive therapy, the preferred SBP range is 120–129 mmHg. Among older individuals (≥65 years), treatment should aim to maintain SBP within the range of 130–139 mmHg. ^e^ In accordance with guidelines [15]: In individuals with diabetes, optimal glycemic control is typically defined as achieving an HbA1c concentration not exceeding 7.0% (53 mmol/mol). For patients with diabetes and elevated cardiovascular risk, LDL cholesterol should be reduced by ≥50% from baseline, with a target value below 70 mg/dL (1.8 mmol/L). In those considered at moderate cardiovascular risk–such as individuals < 35 years of age with type 1 diabetes without chronic complications or additional risk factors, or patients with type 2 diabetes under 50 years of age, diagnosed less than 10 years ago and free from other comorbidities—LDL-C goal is <100 mg/dL (2.6 mmol/L). Additionally, blood pressure should be maintained < 130/80 mmHg.

**Table 2:** Characteristics and functions of galectin-3.

Feature/Function	Description	Ref.
Secretion	Lacks a classical signal sequence; secreted via non-classical pathways (e.g., through exosomes)	[5,8,10]
Role in Apoptosis	Exhibits antiapoptotic activity—binds Bax, Bcl-2, CD95/Fas, stabilizes the mitochondrial membrane, and inhibits cytochrome c release	[25,26,27,28,29,30,31,32,33]
Influence on Proliferation	Overexpression in the cytoplasm promotes tumor growth, while nuclear expression may inhibit cell division	[34,35,36]
Nuclear Function	Plays a role in mRNA splicing and gene expression regulation	[8,37]
Interaction with Receptors	Can stabilize and regulate receptor activity (e.g., EGFR, integrins, immunoglobulins)	[8]
Cell Adhesion and Migration	Enhances intercellular and cell–matrix interactions by crosslinking glycoconjugates	[38,39,40]
Role in Angiogenesis	Stimulates blood vessel formation through interactions with receptors on endothelial cells	[41,42]
Immune Modulation	Binds bacterial LPS, facilitates phagocytosis, and regulates dendritic cell maturation as well as T lymphocyte apoptosis	[4]
Pro-inflammatory Effect	Activates macrophages and neutrophils, and recruits leukocytes	
Anti-inflammatory Effect	May induce T lymphocyte apoptosis and inhibit the secretion of pro-inflammatory cytokines	[5,43,44]
Galectin Network Formation	Oligomerization of gal-3 on the cell surface can organize membrane signaling microdomains via LLPS	[45,46]

Abbreviations: EGFR, epidermal growth factor receptor; LLPS, liquid–liquid phase separation; LPS, lipopolysaccharide.

**Table 3:** The role of galectin-3 in fibrosis and inflammation.

Process	Description of the Mechanism	Ref.
Secretion by macrophages	In response to tissue injury, galectin-3 is released by macrophages and acts in a paracrine manner	[5]
Fibroblast proliferation	It stimulates fibroblasts to transform into myofibroblasts and to increase collagen synthesis	
Impact on the heart	It promotes myocardial fibrosis, leading to increased stiffness and cardiac dysfunction	
Clinical application	A potential biomarker of fibrosis and inflammation, particularly in HF	
Activation of pro-fibrotic pathways	It induces TGF-β, angiotensin II, and endothelin, thereby enhancing fibrosis	[49,50,51]
Inhibition of metalloproteinases	It limits extracellular matrix degradation, promoting collagen deposition	[52,53,54]
Phagocytosis of necrotic cells	It stimulates macrophages to clear damaged tissues, which may help reduce inflammation	[7]
Chronic inflammation	Prolonged exposure to galectin-3 may sustain macrophage activation and chronic inflammation	
Role in cancer	It promotes invasion and metastasis by enhancing cancer cell adhesion	[5,48]
Impact on other organs	Fibrosis in the kidneys, liver, and lungs is involved in the pathogenesis of diabetic nephropathy and idiopathic pulmonary fibrosis	[7,47]
Functioning as a DAMP/alarmin	Galectin-3 signals the presence of tissue damage by stimulating macrophages to release pro-inflammatory cytokines	[4]

Abbreviations: DAMP, damage-associated molecular pattern; HF, heart failure; TGF-β, transforming growth factor beta.

**Table 4:** The impact of tobacco smoking and nicotine on galectin-3 concentrations, molecular mechanisms, and biological effects: data from experimental and clinical studies.

Study Objective	Model of Exposure	Impact on Gal-3 Concentrations	Molecular Mechanisms	Biological Effects Associated with gal-3	Ref.
Smoking vs. gal-3 levels	Population cohort	Weak negative correlation, not significant (*p* = 0.876)	Not evaluated	No independent effect of smoking	[74]
Role of gal-3 in EMT (cigarette smoke)	AECs + CSE (5%)	↑ gal-3, ↑ RUNX-2	ROS → RUNX-2 → gal-3↑; reversed by NAC, GB1107	EMT (↓ E-cadherin, ↑ vimentin), ↑ migration, invasion	[75]
Nicotine and gal-3 in breast cancer	MCF-7 + nicotine (1–100 µM)	↑ gal-3 (up to 5×)	α9nAChR → STAT3 → gal-3↑; mitochondrial stabilization	↑ survival, migration, chemoresistance	[76]
Smoking and gal-3 in COPD	COPD patients during exacerbation/recovery	↑ gal-3 in smokers vs. former/never smokers (*p* < 0.05)	Correlation: hsCRP, pro-BNP; smoking = independent predictor (*β*=0.458)	Inflammation, fibrosis, COPD progression	[77]
CSE and gal-3/autophagy	EPCs + CSE (8%)	↑ gal-3 mRNA (6.7×), protein (5.9×)	ROS → gal-3 → AMPK↑, mTOR↓; reversed by gal-3 shRNA	↑ autophagy, ↓ migration, ↓ angiogenesis	[78]
Gal-3 in MUC1-C/EGFR-dependent EMT	AECs + tobacco smoke	↑ gal-3 in MUC1-C/EGFR complexes	Gal-3 stabilizes MUC1-C/EGFR; glycosylation-dependent	EMT, ↓ E-cadherin, Src/Jnk activation	[79]
Waterpipe smoke and gal-3	Rats exposed to waterpipe smoke	↑ gal-3 in aorta (*p* < 0.001)	↑ TNF-α, IL-1β, VCAM-1, ICAM-1, NF-κB activation, ↓SIRT1	Inflammation, endothelial dysfunction, DNA damage	[82]
Nicotine, DEX and gal-3	Rats + nicotine (0.5 mg/kg)	↑ gal-3 in lung (*p* < 0.001)	↑ IL-1β, IL-6, MDA, TOS; ↓ SOD, GSH-Px; DEX reduces effect	Inflammation, oxidative stress, alveolar damage	[81]

Abbreviations: AECs, airway epithelial cells; AMPK, AMP-activated protein kinase; BNP, B-type natriuretic peptide; COPD, chronic obstructive pulmonary disease; CSE, cigarette smoke extract; DEX, dexpanthenol; EMT, epithelial–mesenchymal transition; EPCs, endothelial progenitor cells; GSH-Px, glutathione peroxidase; hsCRP, high-sensitivity C-reactive protein; ICAM-1, intercellular adhesion molecule-1; IL-1β, interleukin-1 beta; IL-6, interleukin-6; MDA, malondialdehyde; MUC1-C/EGFR, Mucin 1 C-terminal subunit/Epidermal Growth Factor Receptor; NAC, N-acetylcysteine (antioxidant); NF-κB, nuclear factor kappa-light-chain-enhancer of activated B cells; ROS, reactive oxygen species; RUNX-2, Runt-related transcription factor 2; SIRT1, sirtuin 1; SOD, superoxide dismutase; STAT3, Signal Transducer and Activator of Transcription 3; TNF-α, tumor necrosis factor alpha; TOS, total oxidative status; VCAM-1, vascular cell adhesion molecule-1; α9nAChR, α9 nicotinic acetylcholine receptor; ↑, increase; ↓, decrease.

**Table 5:** The impact of various dietary interventions on galectin-3 concentrations and associated inflammatory pathways: a summary of experimental and clinical studies.

Research Model/Population	Type of Dietary Intervention	Study Objective	Effect on Gal-3 and Related Pathways	Ref.
Mice on HFD + HepG2 cells	HFD; palmitic acid	Effect of HFD on gal-3, TLR4, NLRP3 in NASH	HFD: ↑ gal-3, TLR4, NLRP3; ↑ IL-1β, TNF-α, IL-6; suppression: β-lactose, TAK-242	[83]
Rats fed HFD	HFD + MCP (gal-3 inhibitor)	Role of gal-3 in cardiac lipotoxicity	HFD: ↑ gal-3 (heart); MCP ↓ TG, LPC, oxidative stress, mitochondrial damage	[85]
Lgals3^−/−^ mice on atherogenic diet	High-fat, high-cholesterol diet	Role of gal-3 in NASH pathogenesis	gal-3 absence → ↓ steatosis, inflammation, fibrosis; ↓ ALEs	[84]
Wistar rats on HCD	HCD (fats, sugars, salt)	Cardiac changes and gal-3 expression	HCD: ↑ gal-3; correlates with fibrosis, hypertrophy, inflammation	[86]
LGALS3^−/−^ vs. WT mice on HFD	HFD	gal-3 role in adipose metabolism, insulin resistance	gal-3 deficiency: ↑ inflammation, insulin resistance; ↑ M1 macrophages, VAT	[87]
Animal models, literature review	Western diet, processed meat	Effect of dietary elastin on vascular remodeling	Elastin peptides may activate gal-3 pathways; no data for healthy diet	[88]
67 participants (IF vs. control)	IF	Changes in gal-3 and metabolic markers	IF: ↑ gal-3; improved HOMA-IR, ↓ glucose, insulin	[89]
Mice on HFD ± IF	IF vs. HFD	IF effect on gal-3 and WAT inflammation	IF: ↓ gal-3 (serum, WAT); ↓ M1 macrophages, ↓ crown-like structures; ↑ insulin sensitivity	[90]
		IF effect on gal-3 in liver	IF: ↓ gal-3 (liver); ↓ inflammation, macrophage activation, fibrosis (via LCN2, STAT3)	[91]

Abbreviations: ALEs, advanced lipoxidation end-products; Gal-3, galectin-3; HCD, hypercaloric diet; HFD, high-fat diet; IF, intermittent fasting; IL, interleukin; LCN2, lipocalin-2; LPC, lysophosphatidylcholine; MCP, modified citrus pectin; NASH, non-alcoholic steatohepatitis; STAT3, signal transducer and activator of transcription 3; TG, triglycerides; TLR4, toll-like receptor 4; TNF-α, tumor necrosis factor alpha; VAT, visceral adipose tissue; WAT, white adipose tissue; WT, wild type; HOMA-IR, Homeostasis Model Assessment of Insulin Resistance; ↑, increase; ↓, decrease.

**Table 6:** Impact of different forms of physical activity on galectin-3 concentrations in the context of cardiovascular health: a summary of literature data.

Population/Model	Type of Physical Activity	Study Objective	Effect on gal-3	Conclusions	Ref.
Healthy, trained men (runners, cyclists)	Acute HIIT sessions	Assessment of the effect of HIIT on gal-3 concentration and endothelial markers	↑ gal-3 by 39.5%; correlation with circulating endothelial cells	↑ gal-3 as a response to endothelial stress; potential fibrosis-related mechanism	[93]
Amateur marathon runners	Marathon running	Evaluation of changes in cardiac biomarkers	↑ gal-3 after the run (from 8.53 to 10.65 ng/mL), returned to baseline after 2 weeks	Transient ↑ gal-3 as an adaptive response	[94]
Trained runners	Half-marathon	Dynamics of cardiac biomarkers	↑ gal-3 by 33%, normalized within 24 h	↑ gal-3 reflects adaptation, not cardiac injury	[95]
Marathon runners, ultramarathon runners, 10 km runners	Running 42/67/10 km	Dynamics of gal-3 and ST2 after exercise	↑ gal-3 after exertion, returned to baseline within 3 h	↑ gal-3 after running but quickly returns to baseline	[96]
Amateur marathon runners	Marathon running	Assessment of changes in RV function and biomarkers	↑ gal-3 after the run; correlated with ↓ RVEF and VO2max	gal-3 as a marker of cardiac stress and reduced performance	[97]
Mice (VCID model)	3 h of daily activity	Assessment of neuroinflammatory mechanisms involving gal-3	↓ gal-3 expression in white matter	Exercise reduces neuroinflammatory gal-3 expression	[98]
TGF-β1^+/+^ mice on a HCD	Aerobic exercise	Effect of exercise and statins on neuroinflammation	↓ gal-3 expression in microglia	Exercise ↓ gal-3-related neuroinflammatory pathways	[99]
Patients with CAD after COVID-19	8 weeks of HIIT or combined exercise	Evaluation of changes in inflammatory and metabolic markers	↓ gal-3 in both training groups	Exercise ↓ gal-3; combined training is more effective	[100]
Postmenopausal women	HIIT vs. MIACT over 8 weeks	Gal-3 gene expression and lipid profile	↓ gal-3 gene expression (HIIT: −94%, MIACT: −85%)	Physical activity ↓ gal-3 expression; HIIT shows stronger effect	[101]
Patients with CHF and healthy individuals	No physical intervention	Association of gal-3 with physical performance	↑ gal-3 in patients with CHF; inverse correlation with SPPB and hand grip strength	↑ gal-3 concentration associated with poorer physical performance	[102]
Older adults, physically active vs. inactive	Daily physical activity	Assessment of differences in salivary biomarkers	↑ gal-3 binding protein in physically active individuals	Findings relate to LGALS3BP, not gal-3 itself	[103]
Trained athletes	60 km ultramarathon	Dynamics of cardiac biomarkers	↑ gal-3 ×2.4; decreased after 1 h	Transient ↑ gal-3 does not indicate permanent damage	[104]
Patients with CHF with LVEF ≤ 45%	4–6 months of CR	Evaluation of changes in biomarkers after CR	↓ gal-3 by 6.3%	CR ↓ gal-3; confirms anti-inflammatory effect of exercise	[105]
Patients with CHF with LVEF ≤ 40%	12 weeks of aerobic training	Assessment of the effect of gal-3 on training response	↑ gal-3 = no improvement in VO_2_ peak; ↓ gal-3 = improvement in VO_2_ peak	↓ gal-3 predisposes to better training response	[106]
Marathon runners + mouse model	30 km run + animal experiment	Source of gal-3 after exercise	↑ gal-3 after the run; expression primarily in skeletal muscle	gal-3 originates mainly from skeletal muscle, not the heart—clinically important for interpretation	[107]

Abbreviations: CAD, coronary artery disease; CHF, chronic heart failure; CR, cardiac rehabilitation; Gal-3, galectin-3; HCD, high cholesterol diet; HF, heart failure; HIIT, high-intensity interval training; LGALS3BP, Galectin-3 binding protein; LVEF, left ventricular ejection fraction; MIACT, moderate-intensity aerobic continuous training; RVEF, right ventricular ejection fraction; RV, right ventricular; SPPB, short physical performance battery; TGF-β, transforming growth factor beta; VCID, vascular cognitive impairment and dementia; VO_2_, oxygen uptake; ↓, decrease; ↑, increase.

**Table 7:** Galectin-3 dependence on BMI and obesity components: a review of selected population-based and clinical studies.

Study Objective	Study Type and Population	Quantitative Findings (Association with BMI/WC/VAT)	Conclusions Regarding Gal-3 and BMI/Obesity Relationship	Ref.
Assessment of the relationship between gal-3 and metabolic and sleep parameters	Cross-sectional; Chinese population, *n* = 904	BMI: *R* = 0.07, *p* = 0.03; *β* = 0.04, *p* = 0.15; WC: *R* = 0.19, *p* < 0.001; *β* = 0.12, *p* = 0.005	Stronger association with abdominal obesity than with overall BMI	[108]
Profile of gal-1 and -3 in relation to adipogenesis and insulin resistance	Cross-sectional; *n* = 502, POEM study, Sweden	Gal-3: *β* = 0.07, 95% CI: −0.01–0.16; *p* = 0.095	Gal-3 does not significantly correlate with BMI or visceral/subcutaneous fat	[113]
Association between BMI, risk of HF, and gal-3	Prospective cohort; *n* = 8687, ARIC, USA	*OR* for gal-3 ≥ 75th percentile: 2.32 (95% CI: 1.88–2.86; *p* < 0.001) for BMI ≥ 35	Strong association between BMI and elevated gal-3; significantly increased HF risk	[111]
Gal-3 and inflammatory markers in individuals after bariatric surgery	Interventional; *n* = 100, Turkey; assessment 0–6 months post-surgery	BMI vs. gal-3: *r* = 0.375, *p* < 0.001; pre-surgery: 17.6 vs. 14.1 ng/mL (*p* = 0.016)	Gal-3 elevated in individuals with BMI ≥ 40; no reduction after surgery	[112]
Evaluation of cardiac biomarkers based on BMI and HF risk	Prospective cohort; *n* = 8202, PREVEND, Netherlands	Gal-3: 11.7 vs. 10.2 ng/mL (BMI ≥ 30 vs. < 25); *p* < 0.001	Gal-3 increases with BMI but does not independently predict HF compared to other markers	[110]
Sources of gal-3 synthesis; association with T2DM and adipose tissue	Cross-sectional + tissue analysis; *n* = 83, Germany	BMI vs. gal-3: *r* = 0.357, *p* = 0.001; VAT > SAT; PVS > SVS > HVS	VAT is the main site of gal-3 synthesis; correlation with IL-6, leptin, and resistin	[109]

Abbreviations: ARIC, Atherosclerosis Risk in Communities; BMI, body mass index; CI, confidence interval; Gal-3, galectin-3; HF, heart failure; HVS, hepatic vein serum; IL-6, interleukin 6; OR, odds ratio; POEM, Prospective Investigation of Obesity, Energy and Metabolism; PREVEND, Prevention of Renal and Vascular End-stage Disease; PVS, portal vein serum; SAT, subcutaneous adipose tissue; SVS, systemic venous serum; T2DM, type 2 diabetes mellitus; VAT, visceral adipose tissue; WC, waist circumference.

**Table 8:** Overview of biological mechanisms involving galectin-3 leading to vascular dysfunction and elevated blood pressure.

Biological Mechanism/Process	Clinical or Physiological Consequence	Type of Evidence/Research Model	Ref.
↑ gal-3 in response to pro-inflammatory stimuli (angiotensin II, oxidized LDL, advanced glycation end products, interleukin-1β)	Activation of the inflammatory cascade and vascular remodeling	In vivo model (murine), in vitro	[119,120]
Activation of the Src pathway → YAP → gal-3 expression in the endothelium	Promotion of endothelial dysfunction, disturbances in vascular tone	HUVECs, murine model of hypertension	[119]
↓ eNOS, ↓ NO, ↑ ROS, ↑ NADPH oxidase subunits NOX2/p47phox	Impaired vascular relaxation, oxidative stress, endothelial injury	Animal model + cellular studies	
↑ Expression of VCAM-1, IL-6, CD68	Localized vascular inflammation, recruitment of immune cells	Murine model + immunohistochemistry	
Interaction of gal-3 with integrins, modulation of VEGFR2, angiogenesis	Endothelial phenotype alteration, microcirculatory disturbances	In vitro (HUVEC), Matrigel model	[118,124]
Differentiation of VSMCs into an osteoblast-like phenotype	Vascular calcification, increased vascular stiffness	Studies on cell lines and atherosclerosis models (apolipoprotein E-deficient, Apoe^−^/^−^)	[120,123]
Interactions with the ECM, including hyaluronic acid and fibronectin	Vascular wall remodeling, loss of elasticity	Molecular studies, ECM analysis	
↑ Production of type I and III collagen by VSMCs and myofibroblasts	Fibrosis of the vascular media, wall thickening	In vitro, histological studies of blood vessels	[123]
↑ Arterial stiffness (pulse wave velocity, vascular remodeling)	↑ vascular resistance, ↑ SBP	Clinical and experimental data	[122,123]
↑ Afterload → LVH, HFpEF	Left ventricular remodeling, increased left ventricular mass	ECHO assessment + enzyme-linked immunosorbent assay (ELISA, clinical)	[122]

Abbreviations: CD68, cluster of differentiation 68; ECM, extracellular matrix; ECHO, echocardiographic; eNOS, endothelial nitric oxide synthase; Gal-3, galectin-3; HFpEF, heart failure with preserved ejection fraction; HUVECs, human umbilical vein endothelial cells; IL-6, interleukin-6; LVH, left ventricular hypertrophy; NADPH oxidase 2, nicotinamide adenine dinucleotide phosphate oxidase 2; NO, nitric oxide; oxLDL, oxidized low-density lipoprotein; ROS, reactive oxygen species; SBP, systolic blood pressure; SNP, sodium nitroprusside; VCAM-1, vascular cell adhesion molecule-1; VEGFR2, vascular endothelial growth factor receptor 2; VSMCs, vascular smooth muscle cells; YAP, Yes-associated protein.

**Table 9:** Relationships between glucose concentration and gal-3 concentration in individuals with type 2 diabetes mellitus and in experimental models.

Study Objective	Population/Model	Results Regarding Glucose/HbA1c	Results Regarding Galectin-3	Conclusions Regarding Glucose Concentration	Ref.
Review of the role of gal-3 in diabetic cardiomyopathy	Review article	No quantitative data	Gal-3 increases in diabetes and obesity	Relationship with glucose only suggested	[126]
Correlation of gal-3 with HFpEF in patients with T2DM treated with SGLT2i	102 patients with T2DM	HbA1c 8.5% vs. 8.2%, *p* = 0.39 (no sig. difference)	Increased gal-3 concentration in patients with HFpEF (12.64 vs. 9.82 ng/mL; *p* = 0.012)	Gal-3 is an independent predictor of HFpEF, no correlation with glycemia	[127]
Gal-3 and subclinical cardiac dysfunction in T2DM	121 individuals (T2DM + controls)	No correlation with HbA1c	Higher gal-3 in patients (*p* = 0.003)	Gal-3 is a marker of subclinical changes, not linked to current glycemia	[128]
Gal-3 and vascular complications in patients with T2DM	284 patients with T2DM	HbA1c correlates with gal-3 (*r* = 0.217, *p* = 0.018); FBG–no sig. correlation	Higher gal-3 in the presence of complications	Gal-3 moderately correlates with HbA1c, not with FBG	[129]
Gal-3 and cardiovascular risk and mortality in T2DM	1495 patients with T2DM	Higher HbA1c and glucose in deceased patients	Gal-3 weakly correlates with HbA1c (*r* = 0.06, *p* = 0.04)	Gal-3 is an independent risk predictor, weak correlation with HbA1c	[130]
Gal-3 and metabolic parameters in older adults	Geriatric population	HbA1c *r* = 0.267 (*p* = 0.031); glucose *r* = 0.39 (*p* < 0.0001)	Higher gal-3 in patients with T2DM, lower with metformin use	Gal-3 correlates with glucose and HbA1c, reduced by metformin	[131]
Gal-3 and the risk and development of T2DM	Dallas Heart Study, *n* > 3000	FBG/HbA1c not reported	Gal-3 strongly associated with the presence and incidence of T2DM	Gal-3 is a risk marker for T2DM independent of BMI	[132]
Gal-3 and the HFpEF phenotype in patients with T2DM	216 patients with HFpEF	No data on HbA1c/glucose	Higher gal-3 in patients with diabetes (*p* < 0.001)	Indirect association with hyperglycemia, no quantitative data	[135]
Gal-3 and HbA1c in patients with T2DM	100 patients with T2DM	HbA1c inversely correlated with gal-3 (*r* = −0.323; *p* = 0.001)	Lower gal-3 with better glycemic control and metformin use	Gal-3 may reflect metabolic improvement	[109]
Gal-3 and prediabetes and newly diagnosed T2DM	174 individuals (controls, prediabetes, T2DM)	FBG *r* = 0.787; OGTT 2h *r* = 0.833; HbA1c not reported	Gal-3 progressively increases from healthy individuals to T2DM	Strong correlation with glucose and HOMA-IR, good diagnostic marker	[133]
The role of gal-3 in glucose homeostasis–animal model	Lgal3^−^/^−^ mice vs. WT	Impaired glucose tolerance, ↑FBG, ↓insulin	Absence of gal-3 = impaired insulin secretion and resistance	Gal-3 influences glucose metabolism independently of diet	[134]

Abbreviations: BMI, body mass index; FBG, fasting blood glucose; Gal-3, galectin-3; HbA1c, hemoglobin A1c; HFpEF, heart failure with preserved ejection fraction; HOMA-IR, homeostasis model assessment of insulin resistance; OGTT, oral glucose tolerance test; SGLT2i, sodium-glucose co-transporter 2 inhibitors; T2DM, type 2 diabetes mellitus; WT, wild type.

**Table 10:** Sleep health as a biomarker of sleep quality and obstructive sleep apnea: summary of findings from observational and review studies.

Study Objective	Main Findings	Conclusions Regarding Gal-3	Limitations	Ref.
Sleep quality/duration vs. gal-3 levels	Sleep disturbances correlated with ↑ gal-3 concentration (OR 1.68; 95% CI: 1.05–2.68); no association with sleep duration	Gal-3 reflects the impact of poor sleep quality on inflammatory and neuroinflammatory processes	Cross-sectional design; subjective sleep assessment	[108]
Mechanisms linking gal-3 with OSA and sleep disorders	↑ gal-3 concentration in patients with OSA; gal-3 reduction after CPAP therapy	Gal-3 as a neuroinflammatory mediator and indicator of treatment effectiveness	No quantitative data; narrative review	[136]
gal-3 vs. OSA severity and coronary atherosclerosis	Gal-3 ↑ with OSA severity (*p* < 0.001); predictor of OSA severity (*OR* = 2.329) and atherosclerosis	Gal-3 as a biomarker of chronic inflammation and cardiovascular risk in OSA	No intervention data; cross-sectional design	[137]
gal-3 levels in OSA: sex differences	Significantly ↑ gal-3 concentration in women with moderate/severe OSA (*p* < 0.001); no correlation in men	Gal-3 as a potential indicator of cardiovascular risk in women with OSA	Uneven sex distribution; no causal data	[138]
gal-3 and neurocognitive consequences of OSA, review	Gal-3 associated with inflammation and microglial activation in OSA; gal-3 reduction after CPAP	Gal-3 as a marker of neuroinflammatory consequences of OSA and response to therapy	No quantitative data on sleep duration/quality	[139]
Impact of OSA and CPAP therapy on gal-3 and cognition	↑ gal-3 concentration in patients with OSA; reduction after CPAP; correlation with AHI, hypoxia, and cognitive functions	Gal-3 as a link between sleep, neuroinflammation, and cognitive dysfunction	No data on the direct impact of sleep duration on gal-3	[140]

Abbreviations: AHI, apnea-hypopnea index; CPAP, continuous positive airway pressure; Gal-3, galectin-3; OR, odds ratio; OSA, obstructive sleep apnea; ↑, increase; ↓, decrease.

## Data Availability

Not applicable.

## Abbreviations

The following abbreviations are used in this manuscript:

ACC	American College of Cardiology
AECs	Airway epithelial cells
AHA	American Heart Association
AH	Arterial hypertension
AHI	Apnea-hypopnea index
ALEs	Advanced lipoxidation end-products
AMPK	AMP-activated protein kinase
AOR	Adjusted odds ratio
ARIC	Atherosclerosis Risk in Communities Study
BMI	Body mass index
BNP	B-type natriuretic peptide
BP	Blood pressure
CAD	Coronary artery disease
CD68	Cluster of differentiation 68
CHF	Chronic heart failure
CI	Confidence interval
CKD	Chronic kidney disease
COPD	Chronic obstructive pulmonary disease
CPAP	Continuous positive airway pressure
CR	Cardiac rehabilitation
CSE	Cigarette smoke extract
CVD	Cardiovascular disease
CVH	Cardiovascular health
DAMP	Damage-associated molecular pattern
DCM	Diabetic cardiomyopathy
DEX	Dexpanthenol
ECHO	Echocardiographic
ECM	Extracellular matrix
ECV	Extracellular volume
EGFR	Epidermal growth factor receptor
EMT	Epithelial–mesenchymal transition
eNOS	Endothelial nitric oxide synthase
EPCs	Endothelial progenitor cells
ERC	Elastin receptor complex
ESC	European Society of Cardiology
FAK kinases	Focal adhesion kinase kinases
FBG	Fasting blood glucose
FDA	Food and Drug Administration
FHS	Framingham Heart Study
Gal-3	Galectin-3
GSH-Px	Glutathione peroxidase
HbA1C	Hemoglobin A1c (glycated hemoglobin)
HCD	Hypercaloric diet
HF	Heart failure
HFD	High-fat diet
HFpEF	Heart failure with preserved ejection fraction
HFSA	Heart Failure Society of America
HIIT	High-intensity interval training
HOMA-IR	Homeostasis Model Assessment of Insulin Resistance
HR	Hazard ratio
hsCRP	High-sensitivity C-reactive protein
HUVECs	Human umbilical vein endothelial cells
HVS	Hepatic vein serum
ICAM-1	Intercellular adhesion molecule-1
IF	Intermittent fasting
IL-1β	Interleukin-1 beta
IL-6	Interleukin-6
LCN2	Lipocalin-2
LE8	Life’s Essential 8
LGALS3BP	Galectin-3 binding protein
LLPS	Liquid–liquid phase separation
LPC	Lysophosphatidylcholine
LPS	Lipopolysaccharide
LS7	Life’s Simple 7
LV	Left ventricle
LVEF	Left ventricular ejection fraction
LVH	Left ventricular hypertrophy
MCP	Modified citrus pectin
MDA	Malondialdehyde
MIACT	Moderate-intensity aerobic continuous training
MRI	Magnetic resonance imaging
MUC1-C/EGFR	Mucin 1 C-terminal subunit/Epidermal Growth Factor Receptor
NAC	N-acetylcysteine
NADPH oxidase 2	Nicotinamide adenine dinucleotide phosphate oxidase 2
NASH	Nonalcoholic steatohepatitis
NF-κB	Nuclear factor kappa-light-chain-enhancer of activated B cells
NO	Nitric oxide
NWGR	Asparagine-Tryptophan-Glycine-Arginine
NT-proBNP	N-terminal pro–B-type natriuretic peptide
OGTT	Oral glucose tolerance test
OR	Odds ratio
OSA	Obstructive sleep apnea
oxLDL	Oxidized low-density lipoprotein
PI3K	Phosphoinositide 3-kinase
POEM	Prospective Investigation of Obesity, Energy and Metabolism
PREVEND	Prevention of Renal and Vascular End-stage Disease
PUFA	Polyunsaturated fatty acids
PVS	Portal vein serum
Ref.	References
ROS	Reactive oxygen species
RUNX-2	Runt-related transcription factor 2
RV	Right ventricular
RVEF	Right ventricular ejection fraction
SANRA	Scale for the Assessment of Narrative Review Articles
SAT	Subcutaneous adipose tissue
SBP	Systolic blood pressure
SGLT2i	Sodium-glucose co-transporter 2 inhibitors
SIRT1	Sirtuin 1
SOD	Superoxide dismutase
SNP	Sodium nitroprusside
SPPB	Short physical performance battery
SR proteins	Serine/arginine-rich proteins
STAT3	Signal Transducer and Activator of Transcription 3
STEMI	ST-elevation myocardial infarction
SVS	Systemic venous serum
T2DM	Type 2 diabetes mellitus
TC	Total cholesterol
TG	Triglycerides
TGF-β	Transforming growth factor beta.
TLR4	Toll-like receptor 4
TNF-α	Tumor necrosis factor alpha
TOS	Total oxidative status
VAT	Visceral adipose tissue
VCAM-1	Vascular cell adhesion molecule-1
VCID	Vascular cognitive impairment and dementia
VEGFR2	Vascular endothelial growth factor receptor 2
VO2	Oxygen uptake
VSMCs	Vascular smooth muscle cells
WAT	White adipose tissue
WC	Waist circumference
WONDERFUL	Weekly ONe-Day WatER-only Fasting InterventionaL trial
WT	Wild type
YAP	Yes-associated protein
α9nAChR	α9 nicotinic acetylcholine receptor
↑	Increase
↓	Decrease
